# Do socioeconomic factors drive *Aedes* mosquito vectors and their arboviral diseases? A systematic review of dengue, chikungunya, yellow fever, and Zika Virus

**DOI:** 10.1016/j.onehlt.2020.100188

**Published:** 2020-10-23

**Authors:** Ari Whiteman, Jose R. Loaiza, Donald A. Yee, Karen C. Poh, Alexandria S. Watkins, Keira J. Lucas, Tyler J. Rapp, Lillie Kline, Ayman Ahmed, Shi Chen, Eric Delmelle, Judith Uche Oguzie

**Affiliations:** a Smithsonian Tropical Research Institute, Panama City, Panama; b Instituto de Investigaciones Científicas & Servicios de Alta Tecnología, Edificio 219, Clayton PO 0843–01103, Ciudad del Saber, Panama; c Programa Centroamericano de Maestría en Entomología, Universidad de Panamá, Panama; d School of Biological, Environmental, & Earth Sciences, University of Southern Mississippi, Hattiesburg, MS, United States of America; e Department of Entomology, Pennsylvania State University, University Park, PA, United States of America; f Collier Mosquito Control District, Naples, FL, United States of America; g University of North Carolina School of Medicine, Chapel Hill, NC, United States of America; h Woodward Academy, Atlanta, GA, United States of America; i Institute of Endemic Diseases, University of Khartoum, Sudan; j World Reference Center for Emerging Viruses and Arboviruses, The Institute for Human Infections and Immunity, Department of Microbiology and Immunology, University of Texas Medical Branch, Galveston, TX, United States of America; k Public Health Sciences, University of North Carolina at Charlotte, United States of America; l Geography and Earth Sciences, University of North Carolina at Charlotte, United States of America; m College of Natural Sciences Redeemer's University, Ede Osun State, Nigeria; n African Center of Excellence for Genomics of Infectious Diseases Redeemer's University Ede, Osun State, Nigeria

**Keywords:** *Aedes*-borne diseases, Zika virus, Dengue fever, Yellow fever, Chikungunya, Global health

## Abstract

As the threat of arboviral diseases continues to escalate worldwide, the question of, “What types of human communities are at the greatest risk of infection?” persists as a key gap in the existing knowledge of arboviral diseases transmission dynamics. Here, we comprehensively review the existing literature on the socioeconomic drivers of the most common *Aedes* mosquito-borne diseases and *Aedes* mosquito presence/abundance. We reviewed a total of 182 studies on dengue viruses (DENV), chikungunya virus (CHIKV), yellow fever virus (YFVV), Zika virus (ZIKV), and presence of *Aedes* mosquito vectors. In general, associations between socioeconomic conditions and both *Aedes*-borne diseases and *Aedes* mosquitoes are highly variable and often location-specific. Although 50% to 60% of studies found greater presence or prevalence of disease or vectors in areas with lower socioeconomic status, approximately half of the remaining studies found either positive or null associations. We discuss the possible causes of this lack of conclusiveness as well as the implications it holds for future research and prevention efforts.

## 1 Background

The global proliferation of *Aedes aegypti* (L.) and *Aedes albopictus* (Skuse) into novel regions represents a growing public health hazard due to their capacity of transmitting a variety of arboviral pathogens, including the emerging and re-emerging dengue viruses (DENV), chikungunya virus (CHIKV), yellow fever virus (YFVV), and Zika virus (ZIKV) [1]. These mosquitoes are abundant and particularly important in urban environments [2,3], where they use natural and artificial water-holding containers for rearing of larvae and feed nearly exclusively on humans [4]. The threat of vector-borne diseases has risen in recent decades due to the growth of cities, progression of climate change, and increase in globalization and international travel [5]. To date, few control techniques are sustainable, and recent global invasions of these vectors to new areas may further facilitate the proliferation of emerging viral pathogens [6,7], considerably increasing potential public health impacts. Although the biology, virology, and ecology of the vectors have been studied for decades, more recent research efforts have been made to examine arboviral diseases within the context of socioeconomic determinants of health.

Across both urban and rural environments, infrastructure quality disparities, discriminatory zoning practices, and differential exposure to social stressors place significant financial burdens on some neighborhoods over others [8,9]. Residents in these lower socioeconomic neighborhoods are more likely to be exposed to a wider variety of communicable diseases [[10], [11], [12], [13]], along with greater exposure to social costs, including poverty, institutional racism, crime, violence, isolation, and undesirable environmental conditions such as temperature extremes or weather events [14,15]. In contrast, there are also several examples of negative health outcomes associated with relatively high socioeconomic development. These so-called “Diseases of Affluence” include conditions such as diabetes mellitus, cardiovascular disease, and cancer, and these are largely non-communicable conditions associated with high-risk lifestyles and nutrition of highly developed regions [[16], [17], [18]]. Although numerous vector-borne diseases such as leishmaniasis, onchocerciasis, and Chagas disease have been labelled as “Diseases of Poverty” due to their strong association with socially vulnerable populations [19], to our knowledge the association between socioeconomic disparities and diseases caused by DENV, CHIKV, YFV, and ZIKV has yet to be examined in a systematic review process. A 2015 review on the relationship between DENV and poverty found only 12 studies on the subject, with inconclusive results regarding a directional effect [20]. Thus, our objective was to establish the first comprehensive review on the under-studied topic of the most common *Aedes-*borne diseases in the context of socioeconomic determinants of health. For each of the four diseases examined, in addition to *Aedes* occurrence, we aimed to describe the extent of negative, positive, or null relationships with socioeconomic factors. Overall, as a highly understudied subject with considerable public health consequence, we intend this review to serve as a catalyst for further investigations at both the local and global scale.

## 2 Methods

We systematically reviewed existing literatures using the Preferred Reporting Items for Systematic Reviews and Meta-Analyses (PRISMA) guidelines [21], starting with a literature search of the PubMed and Web of Science electronic databases on March 1, 2020. We used Boolean search strings for each of the five categories of this overall review analyses: DENV, CHIKV, YFV, ZIKV, and *Aedes* occurrence. For example, for dengue we used the search strings ‘Dengue’ AND ‘Poverty’ OR ‘Income’ OR ‘Socioeconomic’ OR ‘Socio-economic’ OR ‘Social class’ OR ‘Housing’ OR ‘Employment’ OR ‘Unemployment’ OR ‘Education’ OR ‘Community health services’. We repeated this for each disease. For the *Aedes* occurrence category, the first term of the search string was “‘*aegypti*’ OR ‘*albopictus*’” followed by the same socioeconomic search terms described above. No restrictions for the year of publication or geographic region were applied. If a single study was found for multiple searches because it covered multiple categories (i.e., examined both DENV and CHIKV), the study was included in both or more relevant categories, though only the conclusions specific to each category were analyzed (i.e., only the DENV results were analyzed in the DENV category, while the CHIKV results were analyzed in the CHIKV category).

After removing duplicates within each category using the semi-automatic count function in Mendeley [22], we screened the title and abstract of each study to assess basic subject matter relevance. Studies that met basic subject matter relevance of the review were then read in full and either included in the review or excluded based on meeting any one of six exclusion criteria [20]. First, included studies must not be burden of disease or economic studies. Second, they must not be review articles. Third, studies that did not involve actual disease or virus data were excluded. This includes risk modelling or assessment only studies. Eligible studies were required to use laboratory confirmed cases, clinically suspected cases, vector or host seropositivity, or antigen testing. Fourth, studies that did not involve actual socioeconomic data (e.g., housing quality, income, education) were excluded. Fifth, poor quality studies (small sample size, methodological concerns, or poor description of results) were excluded. Sixth, studies where the abstracts did not accurately reflect the conclusions in the full article were excluded. Excluded studies for the *Aedes* occurrence category were the same except that we excluded studies without actual entomological data (e.g., larval surveys, oviposition surveys) instead of excluding studies without actual disease or virus data. Although burden of disease studies and risk assessment studies are important in understanding the scope and scale of vector-borne disease, this review was intended to focus on empirical analysis of a current or past public health threat, rather than economic impact assessments or future disease risk estimations.

## 3 Results

**Fig. 1:**
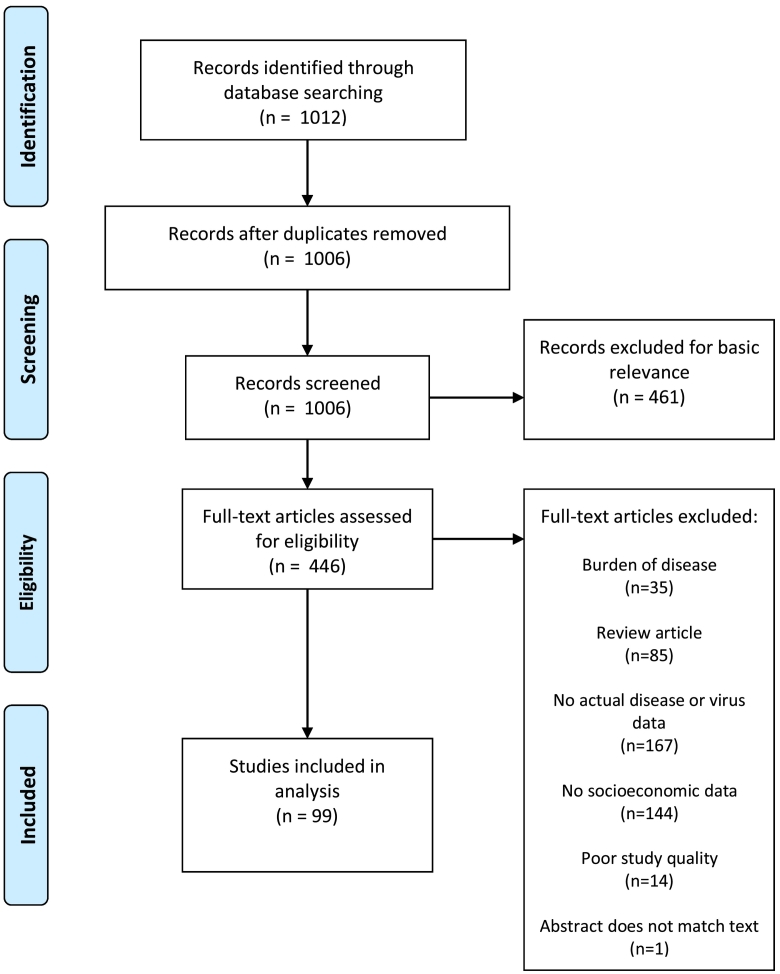
PRISMA flow diagram for DENV.

**Fig. 2:**
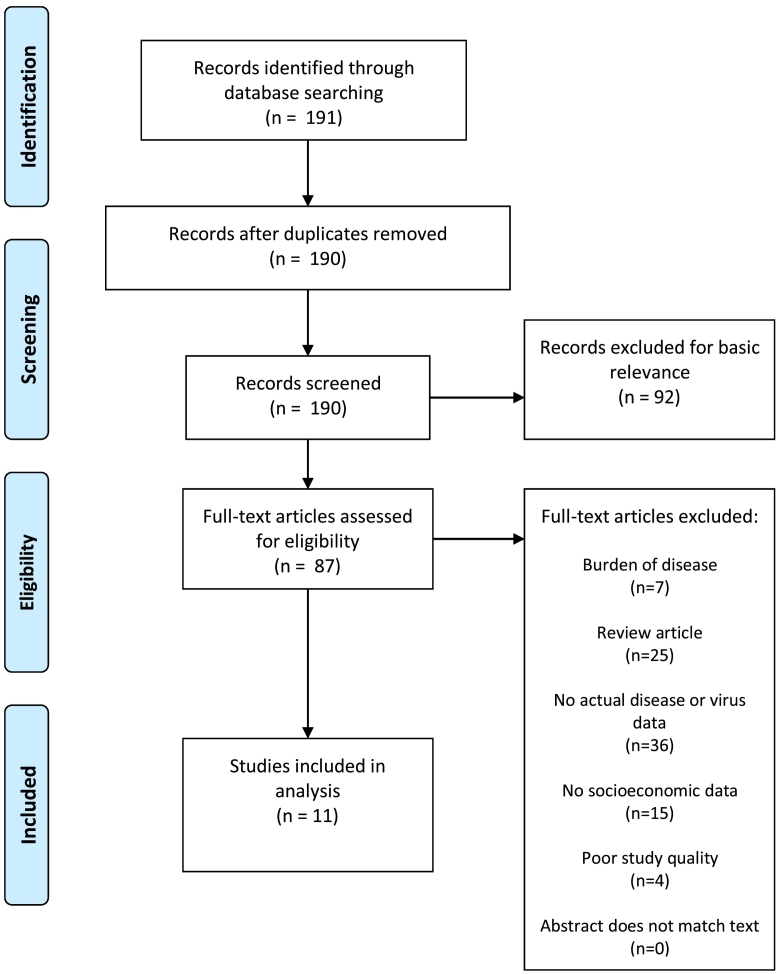
PRISMA flow diagram for CHIKV.

**Fig. 3:**
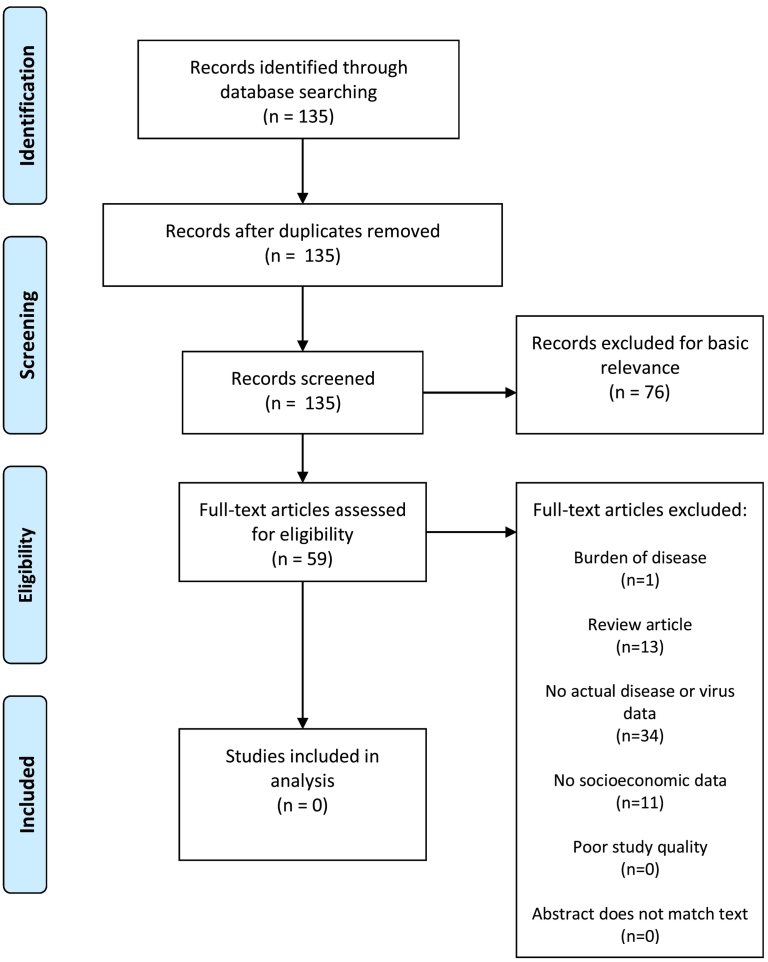
PRISMA flow diagram for YFV.

**Fig. 4:**
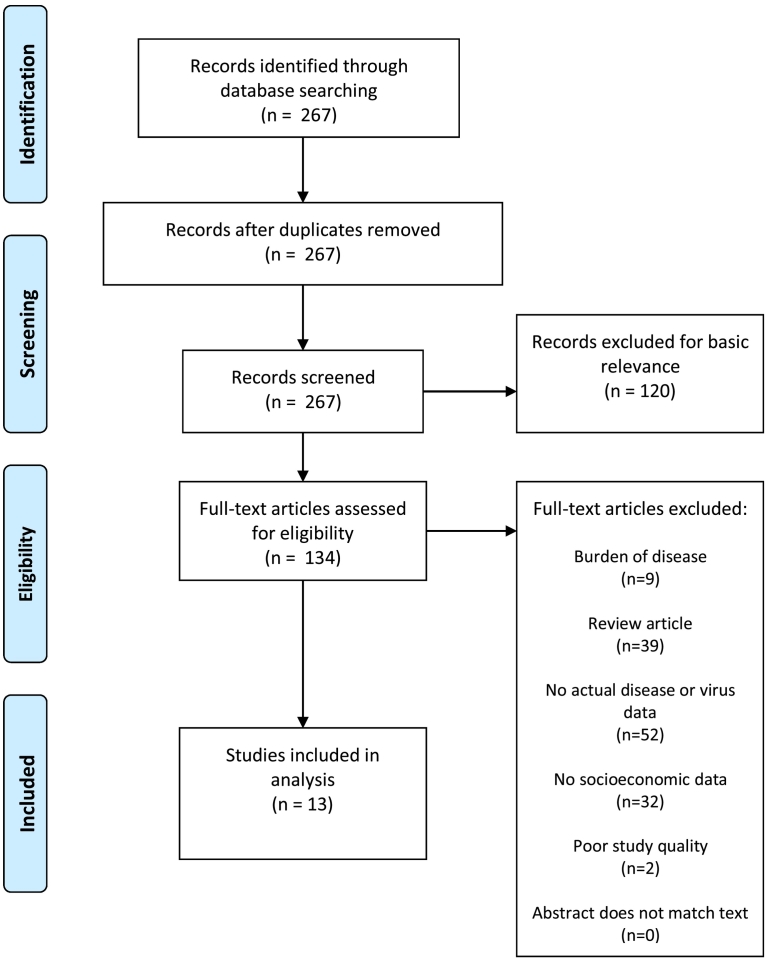
PRISMA flow diagram for ZIKV.

**Fig. 5:**
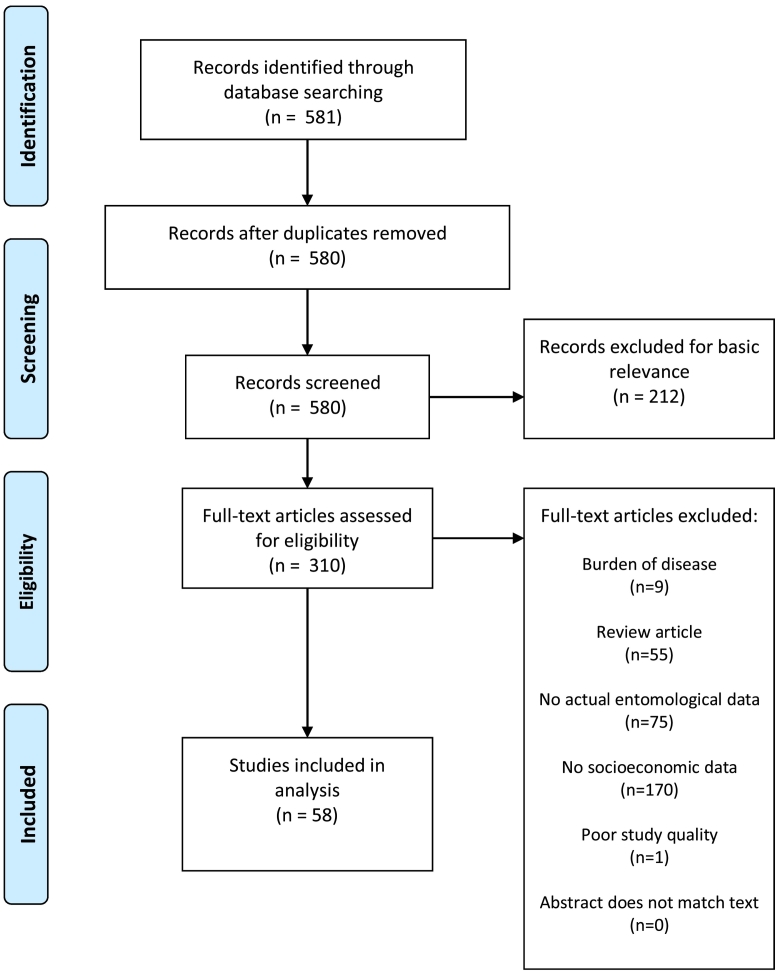
PRISMA flow diagram for *Aedes* presence or occurrence.

The initial search yielded 1012 DENV studies (Fig. 1), 191 CHIKV studies (Fig. 2), 135 YFV studies (Fig. 3), 267 ZIKV studies (Fig. 4), and 581 *Aedes* occurrence studies (Fig. 5). After assessing each category's list of studies against the eligibility criteria, we included 99 DENV studies, 11 CHIKV studies, 0 YFV studies, 13 ZIKV studies, and 59 *Aedes* occurrence studies in the analysis (Appendices). Each study was reviewed for eligibility and analyzed independently by two reviewers. In addition to describing the country of study and socioeconomic metrics used, we determined the directional effect between socioeconomic factors and DENV/CHIKV/ZIKV or *Aedes* occurrence. There were negative (e.g., high disease rates or vector occurrence in low income areas), positive (e.g., high disease rates or vector occurrence in high income areas), or null (e.g. no association found between disease rates or vector occurrence) effects. Some studies had contrasting (i.e., more than one directional effect) or mediated conclusions (e.g., higher disease rates among children in areas of low income, yet higher disease rates among adults in areas of high income). In these circumstances, we further specified three additional directional effects: negative and positive, negative and null, or positive and null. For categories with no zeros values (i.e., having at least a study in every directional effect outcome), we ran a χ^2^ goodness-of-fit test to compare the distribution of effect directions.

**Fig. 6:**
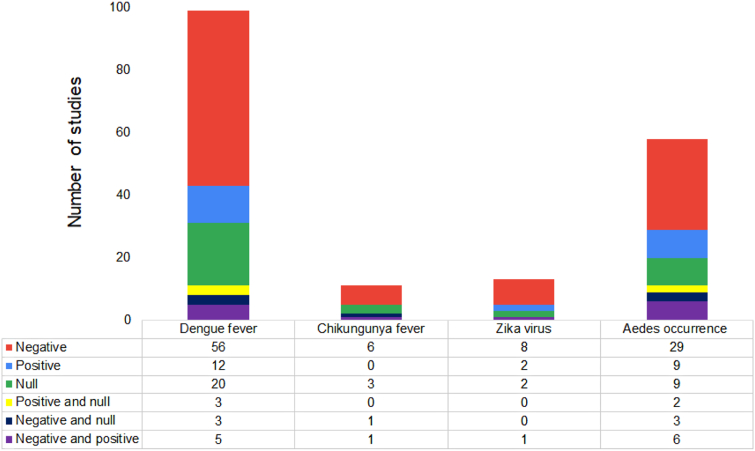
Number and directional effect of studies that empirically examine the relationship between socioeconomic metrics and either dengue fever, chikungunya fever, Zika virus, or *Aedes* occurrence. No studies on yellow fever met the eligibility criteria. A negative effect meant high disease rates or *Aedes* occurrence in low socioeconomic status areas, a positive effect meant high disease rates or *Aedes* occurrence in high socioeconomic status areas, and a null effect meant no association was found between socioeconomic factors and the disease or vector outcomes. (For interpretation of the references to colour in this figure legend, the reader is referred to the web version of this article.)

**Fig. 7:**
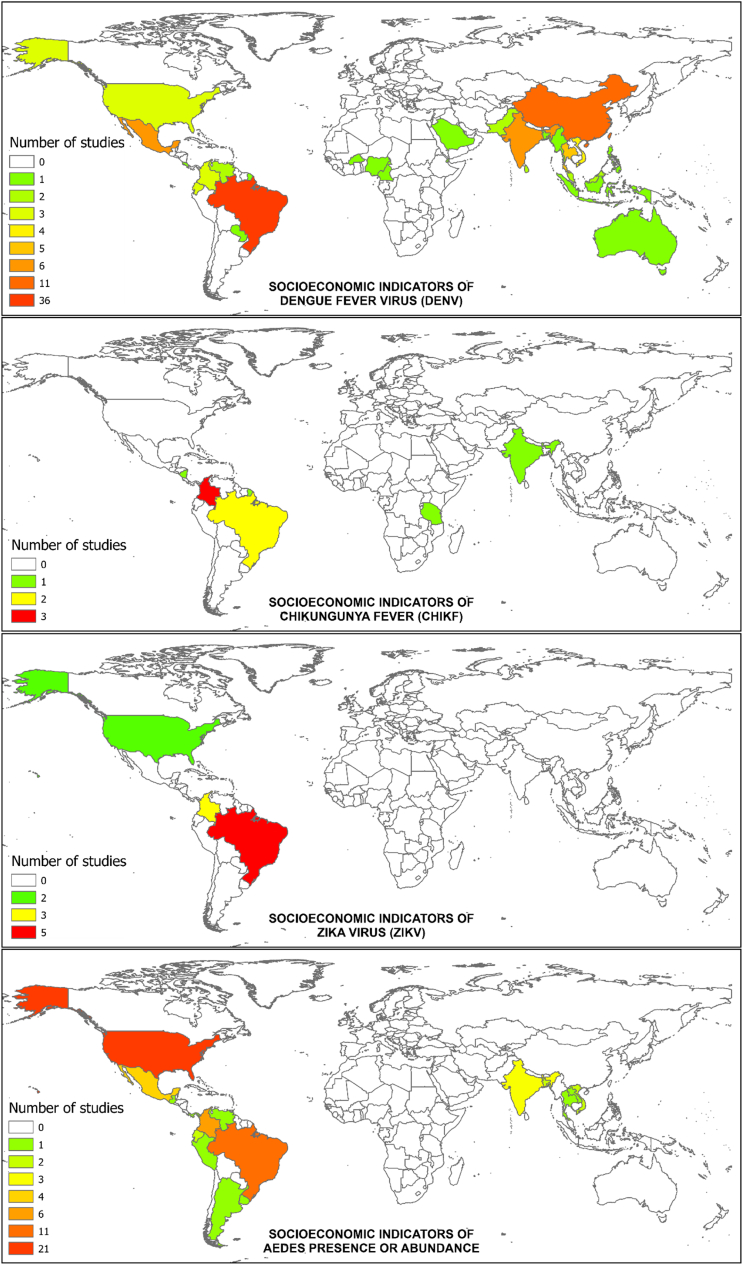
Number of studies included for each country and category in the analysis.

A total of 182 articles were included in the final analysis (Fig. 6). Of the 182 studies, the most (54.39%) were in the DENV category, followed by ZIKV and CHIKV. There were no articles that met the inclusion criteria in the YFV category. For the DENV, CHIKV, and ZIKV categories, Brazil represented the most common location of study, followed by Colombia. For *Aedes*, the United States was the most common setting (Fig. 7).

DENV and *Aedes* occurrence categories had no zero values and the distributions of effect directions were statistically indistinguishable (*P* = 0.70).

### 3.1 Dengue

There were 99 (9.77% of the database search results) DENV studies that met the eligibility criteria. Of these, 36 (52.52%) were conducted in Brazil, followed by 11 (11.11%) in China. The most often used socioeconomic indicator was income, which appeared in 47 (47.47%) of the 99 studies. This was followed by education in 29 (29.29%) studies, and both garbage collection and gross domestic product (GDP) in 11 (11.11%) studies each.

Examples of greater presence or prevalence of DENV in lower socioeconomic areas alone were presented in 56 (56.56%) of the 99 papers, while positive association between DENV and socioeconomic status alone were presented in 12 (12.12%) studies. For papers with multiple contrasting results, five studies (5.05%) found both negative and positive associations between DENV and socioeconomic indicators, while both negative and null results, and both positive and null results were both found in 3 (3.03%) papers, respectively. The frequencies of observed effect directions were significantly different than the expected frequencies based on χ^2^ goodness-of-fit test (*P* < 0.01).

### 3.2 Chikungunya

There were 11 (6.11% of the database search results) CHIKV studies that met the eligibility criteria. Of the included 11 studies, three (25%) were conducted in Colombia, two in Brazil (18.18%), and each of the remaining nine in a separate country. Seven (63.63%) of these included income among their socioeconomic indicators, followed by water access and availability in four (36.36%) studies, housing type and education in three (25%) studies each. Six of the 11 (54.55%) found greater CHIKV prevalence or presence in lower socioeconomic areas, while none of these studies found a positive association. One (9.09%) study found both positive and negative associations, one (9.09%) found both positive and null associations, and three (25%) found no statistically significant association.

### 3.3 Zika

A total of 13 (4.87% of the database search results) studies met the eligibility criteria in the ZIKV category. Eight (72.72%) of the thirteen were conducted in Brazil, while a further three (25%) were conducted in Colombia, and the remaining two came (18.18%) from the United States. Income appeared as a socioeconomic indicator the most, at five (45.45%) studies. Out of the 13 studies, 8 (61.54%) found ZIKV prevalence or occurrence higher in lower socioeconomic areas, while the other two (15.38%) studies found higher ZIKV prevalence or occurrence in high socioeconomic areas. One (7.69%) study found both positive and negative associations and two (15.38%) found no statistically significant association.

### 3.4 Aedes occurrence

A total of 59 (10.15% of the database search results) studies met the eligibility criteria in the *Aedes* occurrence category. Of these, 22 (37.28%) were conducted in the United States, followed by 11 (18.64%) in Brazil, and five (8.47%) in Colombia. The most common socioeconomic indicator was income, which was used in 26 (44.06%) studies, followed by education in 18 (30.50%). Greater occurrence or abundance of *Aedes* mosquitoes in lower socioeconomic areas was demonstrated in 29 (50.00%) studies, while nine (15.52%) studies found a positive association, and nine (15.52%) found a null association. Both negative and positive results were illustrated in six (10.34) studies, whereas both negative and null results were found in three (5.17%) studies, and both positive and null results were found in two (3.45%). The frequencies of observed effect directions were significantly different than the expected frequencies (*P* < 0.01).

## 4 Discussion

Our results indicated a large variability regarding the relationship between socioeconomic factors and the most common *Aedes*-borne diseases and theoccurrence of their most common mosquito vectors. Although the largest share of studies in each category did indeed involve negative associations only, approximately half of the remaining studies included either positive or null associations. This lack of conclusiveness challenges the current practice of labeling DENV, CHIKV, or ZIKV as “Diseases of Poverty,” in a similar manner as malaria, tuberculosis, and HIV/AIDS [23]. Furthermore, approximately one third of the evaluated studies represented the only studies conducted in their respective countries, meaning that in many places the topic remains largely understudied.

There are several possible reasons for the lack of a strong consensus on the directional effect between socioeconomic indicators and either *Aedes-*borne disease or *Aedes* occurrence. The first is that rather than socioeconomic indicators consistently influencing *Aedes-*borne disease or *Aedes* occurrence in a linear relationship across all study sites, the expected effect direction may be highly situationally dependent. This highlights the importance of accounting for socioeconomic variation in any future study on these subjects, considering the bias that may be incurred when conducting research in a socioeconomically homogenous setting. In the absence of studies examining this topic in every threatened region, culture, and across all potential environmental conditions, extrapolations must be drawn from other cases. Based on the results of this review, effectively extrapolating results from other studies and justifying the devotion of limited public health resources may be difficult when the most likely effect globally only occurs approximately 50% of the time.

A second possible explanation for the lack of a singular effect is the simple ubiquity of the vectors and the difficulty in implementing comprehensive vector control. Combating the global spread of vector populations [24] has been difficult due to high resource costs for low budget institutions [[25], [26], [27]], varying effectiveness of the chemical agents [28,29], concerns of their environmental toxicity [30], and the burgeoning issue of insecticide resistance [31]. Even if vector control were to be heterogeneously distributed across socioeconomic gradients [32], the large-scale efficacy of control programs is often limited. Vector hotspots can be as small as 30 m across [33], meaning that even a single missed house in a neighborhood can result in persistent vector populations. In addition, production sources for adults can be extremely cryptic for container-breeding *Aedes* mosquitoes [34,35], making it nearly impossible to identify every possible habitat for larvicide treatment. Furthermore, source reduction of *Aedes* habitat typically requires the integration of the public's involvement [36]; therefore, the efficiency, extent, and variation of vector control educational campaigns may play a role in relationship outcome. To test these hypotheses, we recommend future studies focus on methods and outcomes of vector control between socioeconomically distinct areas. Despite the ubiquity of the vectors and viruses though, the geographic disparities we identified in the literature are striking. Southeast Asia, South Pacific Islands, and sub-Saharan Africa, in particular, represent key gaps in the literature and important areas for future empirical studies to take place.

We also hypothesize that the novelty of pathogens in many regions may limit consistent relationships to socioeconomic factors. With the arguable exception of DENV, which is currently endemic in 125 countries [37], both ZIKV and CHIKV have rapidly spread into new regions within the last decade at the continental scale [7,38,39]. The lack of viral immunity among human hosts would theoretically extend across a socioeconomic gradient, giving each individual human host similar susceptibility, though this does not account for other factors unrelated to the novelty of viruses that influence immunity [40]. Although a small number of the studies in our analysis involved antibody testing, larger scale serosurveillance efforts may help to identify the distribution of immunity across communities with socioeconomic disparities.

Another consequence of ZIKV and CHIKV recently emerging in novel regions is the possible lack of research on their epidemiology. This is evident when comparing the amount of eligible studies in our analysis for ZIKV and CHIKV compared to DENV, the latter of which is more abundant. We expect that in the future more research on socioeconomic determinants of these emerging diseases will be conducted, which can further validate conclusions of this review. The absence of any studies examining the association between socioeconomic indicators and YFV is perhaps not entirely surprising, given it is the only one of the four diseases with a widely available and highly effective vaccine. Subsequently, there have been numerous studies on socioeconomic disparities in YFV vaccine distribution [[41], [42], [43]] instead. However, with around 200,000 cases and 30,000 deaths a year attributed to YFV, 90% being in Africa [44], there is still a strong impetus to examine socioeconomic disparities in actual virus or disease burden. In addition, DENV, CHIKV, and ZIKV are mostly transmitted by urban-dwelling mosquitoes. The impact of socioeconomic variation on host vulnerability may be different when examining vectors and diseases associated more with rural landscapes.

The inability to derive a consensus between socioeconomic indicators and *Aedes*-borne disease or the prevalence of vector mosquitoes from this systematic review is concerning from a public health perspective. Had we established a strong and universal association between *Aedes ­*borne diseases or *Aedes* mosquitoes and socioeconomic indicators, public health resources could be more efficiently allocated towards the most needed communities. The inconclusiveness makes it difficult to determine the risk of *Aedes-*borne diseases across regions, making more people at risk, at least at the global scale. At the local scale, consistency may be easier to establish, which is why repeating the eligible studies in our analysis in different locations is important. As such, a helpful complement to our review may be accomplished by a future review on the types of studies that we specifically excluded, namely burden of disease studies or risk assessments. The burden of disease literature may serve to quantify the larger scale economic and health impacts of socioeconomic disparities in vector or virus occurrence, which may facilitate costs and benefit analyses of interventions. Likewise, a review on risk estimates may extend our conclusions into areas where the vectors and viruses are not present yet may be in the future.

Variation in environmental and cultural practices may have led to contrasting results between continents or regions, meaning that vulnerability in one location may not be comparable to vulnerability in others. While this may be a known component of *Aedes*-borne disease epidemiology, the widespread heterogeneity of the role of socioeconomics in vulnerability had not been previously described. Due to the myriad of variables that influence vector-borne disease risk in addition to socioeconomic conditions among host populations [[45], [46], [47]], a larger scale meta-analysis comparing the types of studies in this review across disparate regions can reveal key variable mediations. For instance, certain socioeconomic indicators may have a greater effect on infection rates only in regions of particular climatic conditions, cultural practices, or levels of public health oversight. This type of analysis will only be possible when the body of literature grows and becomes more evenly distributed across the regions of the world affected by *Aedes*-borne diseases.

## 5 Conclusion

In this review, we demonstrate that socioeconomic indicators of human host communities exhibit inconsistent associations with both *Aedes*-borne diseases and vector distribution. Thus, as further studies are published that explore the important confluence of social vulnerability and vector-borne disease, we emphasize the highly heterogeneous and complex nature of the coupled socio-epidemiological system of *Aedes*-borne diseases across the world. With risk increasing at a faster pace than prevention resources, identifying key socioeconomic indicators in each distinct jurisdiction should be the priority for both the academic and public health teams.

## Declaration of Competing Interest

None.

All authors contributed equally to this study.
